# Polyprenol-Based Lipofecting Agents for In Vivo Delivery of Therapeutic DNA to Treat Hypertensive Rats

**DOI:** 10.1007/s10528-020-09992-9

**Published:** 2020-08-06

**Authors:** Olga Gawrys, Monika Rak, Iwona Baranowska, Sylwia Bobis-Wozowicz, Karolina Szaro, Zbigniew Madeja, Ewa Swiezewska, Marek Masnyk, Marek Chmielewski, Elzbieta Karnas, Elzbieta Kompanowska-Jezierska

**Affiliations:** 1 Department of Renal and Body Fluid Physiology, M. Mossakowski Medical Research Centre, PAS, 5 A. Pawinskiego Street, 02-106 Warsaw, Poland; 2 Department of Cell Biology, Faculty of Biochemistry, Biophysics and Biotechnology, Jagiellonian University, 7 Gronostajowa St., 30-387 Kraków, Poland; 3 Institute of Biochemistry and Biophysics, PAS, 5a A. Pawinskiego Street, 02-106 Warsaw, Poland; 4 Institute of Organic Chemistry, Polish Academy of Sciences, 44/52 M. Kasprzaka Street, 01-224 Warsaw, Poland; 5 Malopolska Centre of Biotechnology, Jagiellonian University, 7 Gronostajowa St., 30-387 Kraków, Poland

**Keywords:** Cationic derivatives of polyisoprenoid alcohols, VEGF-A, Lipofection, SHR, Hypertension

## Abstract

Development of efficient vectors for transfection is one of the major challenges in genetic engineering. Previous research demonstrated that cationic derivatives of polyisoprenoids (PTAI) may serve as carriers of nucleic acids. In the present study, the effectiveness of two PTAI-based formulations (PTAI-6–8 and 10–14) was investigated and compared to the commercial reagents. The purpose of applied gene therapy was to enhance the expression of vascular endothelial growth factor (VEGF-A) in the renal medulla of spontaneously hypertensive rats (SHR) and to test its potential as a novel antihypertensive intervention. In the first part of the study (in vitro), we confirmed that PTAI-based lipoplexes efficiently transfect XC rat sarcoma cells and are stable in 37 °C for 7 days. In the in vivo experiments, we administered selected lipoplexes directly to the kidneys of conscious SHR (via osmotic pumps). There were no blood pressure changes and VEGF-A level in renal medulla was significantly higher only for PTAI-10–14-based formulation. In conclusion, despite the promising results, we were not able to achieve VEGF-A expression level high enough to verify VEGF-A gene therapy usefulness in SHR. However, results of our study give important indications for the future development of PTAI-based DNA carriers and kidney-targeted gene delivery.

**Electronic supplementary material:**

The online version of this article (10.1007/s10528-020-09992-9) contains supplementary material, which is available to authorized users.

## Introduction

Development of efficient and safe vectors for transfection is one of the major challenges in gene therapy (Ramamoorth and Narvekar 2015). Two main classes are currently used: viral vectors and non-viral methods (Nayerossadat et al. 2012). Virus-based vectors are considered more effective, but plagued by safety concerns, due to their immunogenicity and cytotoxicity (Jafari et al. 2012). Several studies indicate that cationic lipids enhance the efficiency of nucleic acids and drug delivery to the cells (Namiki et al. 2011; Jafari et al. 2012). Lipid‐based carriers are known for their safety, versatility, low immunogenicity, simplicity of application, and easy modification for specific conditions (Wang et al. 2013). Cationic lipids interact spontaneously with negatively‐charged nucleic acids and form lipoplexes (complexes of lipids and DNA). Addition of neutral lipids (also called helper or co‐lipids), such as l-*α*-phosphatidylethanolamine dioleyl (DOPE) or cholesterol, change the properties of lipoplexes making them more suitable for some applications. Cholesterol is often suggested as a good helper lipid for in vivo use. Despite the fact that numerous cationic and helper lipids have been synthesized (many of them being the subjects of patent applications (Koynova and Tenchov 2011), none of the developed lipofecting agents appear to be perfect. As a result, intensive research has been carried out aiming to elaborate an optimal lipofecting formulation.

Polyisoprenoid alcohols (polyprenols and dolichols) are long chain, linear polymers composed of isoprene units [from several up to more than 100 units, (Swiezewska and Danikiewicz 2005)] found in almost all living organisms (Skorupinska-Tudek et al. 2008). They are involved in cell response to environmental stress, glycosylation, and prenylation of proteins, and also they intensify the fusion and increase the permeability of model membranes (Swiezewska and Danikiewicz 2005). Recent studies (Gawrys et al. 2014b, 2018; Grecka et al. 2016; Rak et al. 2016; Stachyra et al. 2017) show that cationic derivatives of polyisoprenoid alcohols, called PTAI, are non-toxic and may serve as lipofectants. In the present study, the effectiveness of two PTAI mixtures was investigated for plasmid DNA delivery into rats’ cells and to rats and compared to the commercially available reagents for transfection: in vivo-jetPEI® (Polyplus-transfection) and Avalanche®-in vivo Transfection Reagent (EZ Biosystems LLC).

PTAI-based reagents were used to enhance the expression of vascular endothelial growth factor type A (VEGF-A) in spontaneously hypertensive rats (SHR). Hypertension is a worldwide health problem, because of its high prevalence and associated renal and cardiovascular diseases, such as chronic kidney disease, heart failure, ischemic heart disease, stroke and other cerebrovascular or retinal diseases (Chockalingam et al. 2006; World Health Organization 2013). Despite decades of research, hypertension is still inadequately diagnosed, largely because of incomplete understanding of the underlying pathogenic mechanisms. Recent studies provide an ample evidence supporting an important role of VEGF deficiency in the development of hypertension and related kidney diseases (Bhargava 2009; Chade 2016). VEGF is a principal regulator of various physiological processes including angiogenesis, mediating increased vascular permeability, proliferation, and lymphangiogenesis (Robinson et al. 2010). The first indication of an association of VEGF-A and hypertension was when VEGF inhibitors were applied for anticancer therapy. In all cases of inhibition of the VEGF signaling pathway, hypertension was the most common side effect [currently it occurs in up to 80% of patients treated with these drugs (Bhargava 2009; Robinson et al. 2010)]. The mechanism of hypertension associated with VEGF inhibition is still not clear and it is uncertain what is crucial for the development of suitable therapeutic strategies for patients (Chade 2016).

The aim of presented study was to evaluate if enhanced expression of VEGF-A in the renal medulla—crucial area for renal blood pressure regulation (Cowley 2008) will increase its circulation and reduce blood pressure of hypertensive rats.

## Materials and Methods

### Plasmids

The reporter plasmid pEGFP‐C1 encoding enhanced green fluorescent protein (eGFP) was kindly supplied by Professor A. F. Sikorski (University of Wrocław, Poland). Plasmid encoding rat vascular endothelial growth factor A (rVegf), transcript variant 1, NM_001287107.1 was purchased from OriGene (Rockville, MD, USA). The rVegf cDNA was PCR amplified, purified using GeneMATRIX DNA/PCR Clean-up Purification Kit (Eurx, Gdansk, Poland), and ligated (T4 DNA Ligase; Invitrogen/Thermo Fisher Scientific; Waltham, MA, USA) to plasmid #67275 (a gift from Jannik Elverløv-Jakobsen, Addgene; Watertown, MA, USA) after deletion of TurboFP with PacI and XhoI restriction enzymes. mCherry sequence was PCR amplified from pLV-mCherry #36084 plasmid (RRID**:**Addgene_36084; a gift from Pantelis Tsoulfas, Addgene) and cloned to the #67275-rVEGF using AgeI and ClaI restriction sites, creating pCAG-rVegf-2A-mCherry plasmid. Control plasmid, pCAG-mCherry, containing only mCherry expression cassette was generated by PCR amplification of mCherry and cloning into #67275 plasmid using PacI and ClaI restriction sites.

All amplification reactions were performed using Phusion HF polymerase (Thermo Fisher Scientific) and all restriction enzymes were purchased from New England Biolabs (Ipswich, MA, USA). Plasmid sequences were verified by restriction analysis and DNA sequencing (Genomed, Warsaw, Poland).

### PTAI Chemical Synthesis and Purification

**Fig. 1:**
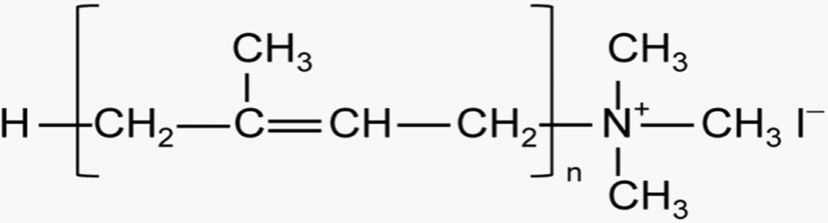
Trimethylpolyprenylammonium iodide (PTAI)

Trimethylpolyprenylammonium iodides (PTAI, Fig. 1) were prepared from naturally occurring polyprenols according to Madeja et al. (2007) with modifications. Two types of PTAI preparations with different length polyprenoid chains were used in this work: PTAI-6–8 and PTAI-10–14, containing a mixture of polyprenols with chain lengths from 6 to 8 or 10 to 14 isoprene units, respectively. All polyprenols used for the synthesis of cationic derivatives were from the Collection of Polyprenols, Institute of Biochemistry and Biophysics PAS, Warsaw. The relevant preparation procedures have been described in a patent (No. 211824, Polish Patent Office 2012). All products were stable as demonstrated by thin layer chromatography on silica gel plates (Merck, Darmstadt, Germany), which showed single spots in chloroform/methanol/water (65:25:4,; Rf = 0.66); in n-butanol/acetic acid/water (66:17:17; Rf = 0.23); in n-propanol/ammonia/water (8:1:1; Rf = 0.10) , and in ethyl acetate/methanol/acetic acid (75:20:5; Rf = 0.03). No symptoms of decomposition were detected for any of the compounds after 1 year of storage in a dry state in argon atmosphere at – 80 °C.

### Preparation of Reagents Based on PTAI

PTAIs (PTAI-6–8 and -10–14) and helper lipids (Sigma-Aldrich, St. Louis, USA) l-*α*-phosphatidylethanolamine dioleyl (DOPE) and 3ß-[*N*-(*N*′,*N*′-dimethylaminoethane)-carbamoyl]cholesterol hydrochloride (DC-cholesterol; DC-CHOL) were dissolved in ethanol (99%). The following two compositions containing PTAI and helper lipids in ethanol at the indicated molar ratios were prepared: PTAI-6,7,8+DOPE—1.5:1 and PTAI-10–14+DOPE+DC-cholesterol—1:1:1. Obtained mixtures were mixed with DMEM F-12 HAM cell culture medium without additions of serum and antibiotics and intensely vortexed for 3 min. Ethanol content in the mixtures was PTAI-6–8/DOPE—11,4% and PTAI-10–14/DOPE/DC-cholesterol—20.7%. The compositions were stored at 4 °C up to 7 days. These compositions were previously shown to be effective DNA vaccine carriers (Stachyra et al. 2017). They (and their use) are the subject of patents (No. 231158, Polish Patent Office 2019; No. 230096, Polish Patent Office 2018) and patent application (No. PCT/PL2015/000093, WO/2016/032348, Polish Patent Office, European Patent Office).

### In Vitro Transfection Efficiency

#### Cell Culture

Rat sarcoma XC cells (ATCC Cat# CCL-165, RRID:CVCL_1891) were cultured in DMEM Low-Glucose medium (Sigma-Aldrich, St. Louis, USA) supplemented with 10% heat-inactivated fetal bovine serum (FBS; Gibco Lab., New York, USA). Media were supplemented with 100 IU/ml penicillin and 10 µg/ml streptomycin (Polfa, Tarchomin, Poland) and cells were cultured in a humidified atmosphere with 5% CO_2_ at 37 °C. For transfection experiments, cells were seeded into the wells of a 24-well plate at a density of 8 × 104 cell/well and cultivated for 24 h in media with 10% FBS without antibiotics (to reach 70–80% confluence).

PTAI-6–8/DOPE and PTAI-10–14/DOPE/DC-cholesterol mixtures were diluted in serum‐free and antibiotic-free cell culture medium or 5% glucose and mixed with pEGFP‐C1 plasmid diluted in cell culture medium or 5% glucose, respectively, to final concentration of 0.2 μg/μl. Next, they were incubated for 30 min at room temperature to obtain lipoplexes and diluted in serum‐free DMEM F12 HAM medium (1:4). 2.5 μg of PTAI- 6–8/DOPE and 4 μg of PTAI-10–14/DOPE were used for transfection of cells per well of 24-well plate. Lipids and pDNA were mixed at calculated ± charge ratio of 1:2.0 for PTAI-10–14 and 1:2.3 for PTAI-6–8-based lipoplexes.

#### The Transfection Efficiency of PTAI in Glucose Medium

The transfection efficiency was calculated after 24 h of cell incubation with lipoplexes. Cells were washed with phosphate-buffered saline (PBS), incubated in the presence of Hoechst 33342 (1 µg/ml in PBS; Sigma-Aldrich, St. Louis, USA) for 10 min, washed again with PBS, and submerged in DMEM Low-Glucose medium with 10% FBS. The percentage of transfected cells was calculated under fluorescent Leica DMI6000B AF7000 microscope (Leica Microsystems GmbH, Wetzlar, Germany) according to the following formula:

$$Le=\left(\frac{NmCh}{NHoe}\right)*100\%$$

where *NmCh* is the number of cells expressing mCherry *and NHoe* is the number of cells stained with Hoechst 33,342.

#### Prolonged Stability of PTAI-Based Lipoplexes

Stability and effectiveness of the PTAI-based lipoplexes after incubation in 37 °C up to 7 days was tested in vitro. Transfection mixtures were prepared as described for osmotic pumps administration (see below). In the serum-free procedure, the medium from cells was aspirated and replaced with 300 µl of serum-free DMEM Low-glucose medium and 100 µl of the lipoplexes suspension containing 3 µg of lipids [FBS(−); serum-free conditions]. In the presence of serum procedure [FBS(+)], 200 µl of medium was left in the well and 100 µl of the lipoplexes suspension was added together with 100 µl of medium without serum and antibiotics. After 5 h of incubation at 37 °C in 5% CO_2_, 400 µl of DMEM Low-Glucose medium supplemented with 20% of FBS and antibiotics was added to the transfection medium.

#### Flow Cytometry Analysis

For flow cytometry analysis, cells were washed with phosphate-buffered saline (PBS) and harvested with 0.25% trypsin with 0.02% EDTA. For GFP reporter analysis, cells were suspended in DMEM low-glucose serum-free medium and acquired on Guava® easyCyte 8 flow cytometer (Luminex, Austin, TX, USA), with 488 nm laser excitation. The percentage of GFP-positive cells was analyzed by InCyte software ver. 3.3 (Luminex, Austin, TX, USA) and presented as average from each group with SEM values.

For mCherry analysis, Amnis ImageStream® X MkII imaging cytometer (Luminex, Austin, TX, USA) was utilized. This unique system allows not only to perform high-throughput, quantitative multi-parameter flow cytometry measurements with high sensitivity, but additionally, based on advanced charge-coupled device (CCD) camera imagery features, it simultaneously collects galleries of single objects in up to 12 channels. Thus, this high-end system combines the speed and statistical significance of flow cytometry with the power of fluorescent microscopy, allowing to perform sophisticated morphometric analyses of signal distribution, on single cell as well as on whole population level. For cell acquisition, INSPIRE® software (Luminex, Austin, TX, USA) was used, with 561 nm laser excitation and ×40 magnification objective. Data were analyzed using IDEAS® software ver. 6.2 (Luminex, Austin, TX, USA).

### In Vivo

#### Preparation of the Transfection Mixtures for In Vivo Experiments

Two PTAI compositions were prepared as described above. Two commercially available reagents were used: Avalanche®-in vivo Transfection Reagent (cat. no EZT-VIVO-1, EZ Biosystems LLC, Maryland, USA) and in vivo-jetPEI® (cat. no 201-10G, Polyplus-transfection S.A, Illkirch, France). The reagents were handled according to the manufacturer’s instructions.

*For intravenous infusion (I),* appropriate amount of plasmid DNA (150–180 µg per intravenous injection, see Table 1 for details) was diluted with ultrapure MiliQ water and 10% sterile glucose to obtain ½ injection volume (5% final glucose concentration). PTAI-10–14-based lipoplexes were prepared at ± charge ratio of 1:1.7. The appropriate amount of reagent for transfection also was diluted with sterile 10% glucose and MiliQ water to achieve ½ injection volume (5% final glucose concentration). Then both solutions were mixed together and incubated for 15–30 min (room temp.).

*For osmotic pumps administration (II),* 100 µg of the nucleic acid was used for one osmotic pump (for pump details see below *Intrarenal infusion with ALZET® Osmotic Pumps*), hence each rat received 200 µg of DNA in total (to both kidneys). The DNA to reagent ratio was as follows: 0.5 µl of PTAI-10–14, 0.6 µl of PTAI-6–8 and 0.14 µl for jetPei®, and Avalanche per 1 µg DNA (± charge ratio of 1:2.0 for PTAI-10–14 and 1:2.3 for PTAI-6–8-based lipolexes). The preparation of the solutions was as described above for intravenous injection. Control group of animals received osmotic pumps filled only with the solvent (sterile 5% glucose without DNA and without any reagent for lipofection). Filled osmotic pumps were started-up by incubation in 37 °C overnight in sterile 0.9% saline before implantation. This step is essential when catheters are used to minimize the chance of an occlusion or clot formation. All preparations were made in sterile conditions in laminar flow cabinet.

#### Animals

The experimental procedures were approved by the I Ethical Committee for Animal Experimentation (Warsaw), which follows the European Directive 2010/63/EU on the protection of animals used for scientific purposes (no 34/2015, 479/2017). Male, adult (16 weeks old, mean body weight 299 ± 4 g) spontaneously hypertensive rats (SHR) bred at the Animal House of Mossakowski Medical Research Centre, Polish Academy of Sciences (*n* = 44) were fed ad libitum a standard diet (STD, 0.25% Na w/w, SSNIFF GmbH, Soest, Germany) and had free access to drinking water during the whole experiment. Animals were housed 2 per cage in a conventional animal room with controlled temperature (24 ± 2 °C) and a 12/12 h light–dark cycle.

#### Protocols

The study was divided into two protocols. The first set of experiments was to choose the optimal conditions for transfection and to evaluate and compare the effectiveness of selected PTAI and commercial reagents for DNA delivery after intravenous administration (I, *n* = 21). In the second phase, we wanted to evaluate the impact of enhanced expression of VEGF-A administered directly into the renal medulla of adult SHR with ALZET® Osmotic Pumps (II, *n* = 23).

#### Intravenous Injection (I)

In the preliminary experiments, various conditions were tested (summarized in Table 1) i.e., the DNA:reagent ratio (from 0.12 to 0.20), number of injections, and duration of the observation (24–72 h). The intravenous infusions to adult SHR were conducted under isoflurane anesthesia (IsoVet®, Piramal Healthcare, UK) at 4% in the induction phase and maintained by mask inhalation at 2–1.5% during the procedure (Combi-vet® system, Rothacher Medical GmbH, Heitenried, Switzerland). Lipofecting solutions (1 ml) containing appropriate reagent for transfection mixed with plasmid DNA were infused via tail vein at a rate of 6 ml/h for 10 min. After awakening from anesthesia, rats were observed for 24, 48, or 72 h (Table 1). Then all rats were anaesthetized with intraperitoneal sodium thiopental (Thipen®, 100 mg/kg; Samarth Life Sciences Pvt. Ltd, Baddi, India) and perfused with PBS. Fragment of the spleen, liver, lung, and whole kidney were collected and kept in ice-cold PBS until flow cytometry measurement (analysis of fluorescence of the reporter protein was performed on the same day). Another fragment of the spleen, liver, lung, heart, brain, and the second kidney were frozen in dry ice for further microscopic analysis (− 80 °C).

#### Intrarenal Infusion with ALZET® Osmotic Pumps (2 Week Observation, II)

SHR (*n* = 23) were implanted with telemetry transmitters (Data Sciences International, St. Paul, USA) for blood pressure measurements. The procedure was conducted under isoflurane anesthesia (see above). After 7 days of recovery, rats were randomly divided into five groups and again under isoflurane anesthesia were implanted with osmotic pumps (model 2001, release rate 1 µl/h, duration 1 week, reservoir volume 200 µl, ALZET® Osmotic Pumps, Cupertino, USA). To each osmotic pump, a catheter was attached connected to ALZET Brain Infusion Kit 2 (ALZET® Osmotic Pumps, Cupertino, USA) allowing the infusion of selected solutions into the renal medulla. Flank incision was made and the kidney was exposed. The 3 mm needle of the infusion kit was inserted into the renal medulla (on the border on the outer and inner medulla of the kidney) and secured with tissue glue (Histoacryl®, Braun Surgical S.A. Rubi, Spain). The incision was closed with sutures and the procedure was repeated for the second kidney (each rat received two mini-osmotic pumps).

Blood pressure (BP) was measured before the implantation of the osmotic pumps (day 0) and on 1, 4, 8, 10, and 14th day of the observation, starting at 3 pm and finishing at 8 am on the next day. Urine samples were collected on days 0, 7, and 14th in metabolic cages (Tecniplast S.p.A. Buguggiate, Italy). After 2 weeks, rats were anaesthetized with intraperitoneal sodium thiopental and perfused with PBS. Half of one kidney was harvested and kept in ice-cold PBS until flow cytometry analysis. Second half was cut with surgical scissors to separate cortex and medulla, which were frozen separately in dry ice. Whole second kidney was harvested and frozen on dry ice. All frozen tissues were stored in − 80 °C until further analysis (ELISA, microscopic).

#### Tissue Homogenization and Flow Cytometry

Tissues were weighted and dissected with surgical scissors into 1 mm pieces and 3 ml of preheated (37 °C) collagenase IV (0.15 PZ U/ml, SERVA Electrophoresis GmbH, Heidelberg, Germany) with 2 mM calcium chloride solution added to each sample. Tissues were incubated in 37 °C on rocking platform with gentle agitation (spleens for 10 min, kidneys for 15 min, livers for 30 min and lungs for 40 min). The enzymatic digestion was stopped by putting the solution on ice. Afterwards each solution was carefully pipetted back and forth for 15–20 times and filtered through 70 µm nylon cell strainer, followed by centrifugation (300×*g*, 4 min). The supernatant was discarded and cells were suspended in RBC lysing buffer for red blood cell removal. After 10 min incubation (room temp.), cells were centrifuged (300×*g*, 4 min) and washed with FCM buffer. The fluorescence of reporter proteins (eGFP or mCherry) in obtained single cells suspension was analyzed on BD FACS Canto II flow cytometer (BD Biosciences, Poland). Just before the analysis, the cells were filtered again using Flowmi Cell Strainers for 1000 µl Pipette Tips (porosity 40 µm, Bel-Art—SP Scienceware, Wayne, USA).

The results were acquired and analyzed with Diva software (BD Biosciences, Poland). Forward scatter area (FSC-A) *versus* side scatter area (SSC-A) density plots were used for gating to exclude debris and identify cells of interest based on the size and granularity (gate P1). To exclude doublets, a forward scatter height (FSC-H) *versus* forward scatter area (FSC-A) density plots were constructed and single cells were gated (gate P2). Single parameter histograms were used to further identify distinct cells that express a reporter gene (eGFP or mCherry).

#### VEGF-A Concentration in Urine and Renal Homogenates

VEGF-A protein level was measured with commercial ELISA kit (cat no K5365, BioVision, Inc., San Francisco, USA) with detection range 15.6–1000 pg/ml according to the manufacturer’s instruction. Kidneys (cortex and medulla separately) were weighted and mechanically homogenized in glass homogenizers in RIPA buffer (1 mg of tissue for 2 µl of the buffer, Sigma-Aldrich, Poland) with proteases inhibitors (cOmplete™, Mini Protease Inhibitor Cocktail, Sigma-Aldrich, Poland). Renal homogenates were diluted 1:200, whereas urine was diluted 1:5 with provided sample diluent buffer. Total protein content in renal homogenates was measured with Pierce™ BCA Protein Assay Kit (Thermo Fisher Scientific, Poland) according to the attached instructions.

### Chemicals and Buffers

*RBC lysis buffer* (10× concentration) for red blood cells removal was prepared by mixing 8.02 g of ammonium chloride (NH_4_Cl), 0.84 g of sodium bicarbonate (NaHCO_3_), and 0.37 g of EDTA (disodium) and the volume was adjusted to 10 ml with MiliQ water. Stock solution was stored at 4 °C and diluted (1:10 with MiliQ water) on the day of the analysis.

*FCM buffer* for flow cytometry analysis was prepared by mixing 2.5 g albumin fraction V (AppliChem GmbH, Darmstadt, Germany), 0.16 g EDTA (disodium), and 0.5 g sodium azide (NaN_3_) and the volume was adjusted to 500 ml with PBS. The solution was stored at 4 °C for 6 months.

### Statistics

Data are presented as the means ± SEM. The significance of changes was evaluated by repeated measures multivariate analysis of variance (ANOVA), followed by a post hoc Duncan’s multiple range test. For some parameters, one-way ANOVA was used. In cases where the data did not meet the assumptions for ANOVA, non-parametric tests were used (Kruskal–Wallis H test). All calculations were mad with STATISTICA software (version 10.0, StatSoft, Inc. Poland). The level of statistical significance was set at *p* < 0.05.

## Results

### In Vitro

**Fig. 2:**
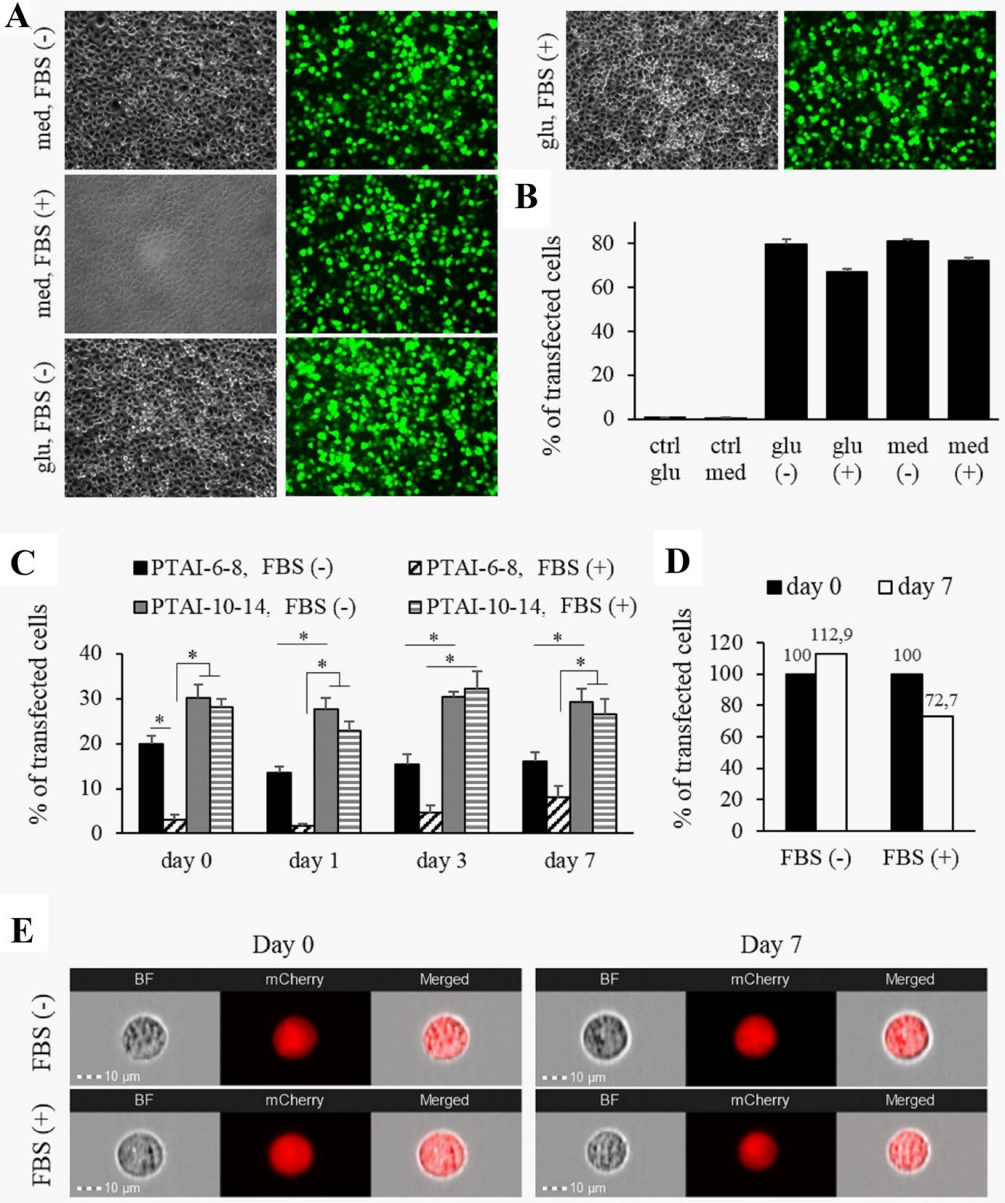
Efficiency of DNA transfer into XC cells in the presence (FBS+) and absence (FBS-) of serum with PTAI-based lipoplexes. **a**, **b** XC cells transfected with PTAI-10–14+DOPE+DC-cholesterol using pEGFP-C1 plasmid, lipofecting mixtures prepared in cell culture medium (med), or 5% glucose solution (glu) and used in the absence FBS (−) or presence FBS (+) of serum, **a**—representative images, **b**—efficiency of lipofection evaluated by flow cytometry; each value represents the mean ± SEM (*n* = 6); **c–e** XC cells transfected with PTAI-6–8+DOPE and PTAI-10–14+DOPE+DC-cholesterol using mCherry-VEGF-A plasmid with lipoplexes prepared in 5% glucose and stored at 37 °C for 1–7 days (day 0—lipoplexes used after preparation, not stored, days 1–7—lipoplexes stored for 1–7 days); **c**—efficiency of lipofection counted using fluorescence microscope, each value represents the mean ± SEM (*n* = 3); **d**—no loss of efficiency of PTAI-10–14-based lipoplexes, data shown as % of control on day 0 evaluated with ImageStream® X MkII system; **e**—representative images of cells obtained based on the ImageStream® X MkII analysis at day 0 and day 7. Exemplary galleries of cells in bright field (BF) and mCherry fluorescence channel were presented; **a**–**e** molar ratios: PTAI-6–8+DOPE—1.5:1 and PTAI-10–14+DOPE+DC-cholesterol—1:1:1, PTAI+DOPE concentration: PTAI-6–8-based lipoplexes—4 µg/well of 24-well plate, PTAI-10–14-based lipoplexes—4 µg/well of 24-well plate, DC-cholesterol added as additional lipid at indicated molar ratio; * *p* < 0.05, non-parametric Kruskal–Wallis H test

In our previous studies we have shown that cationic polyisoprenoids are effective components of lipofecting mixtures (Rak et al. 2016) and DNA vaccines vehicles (Stachyra et al. 2017). In the present study, we have tested PTAI-6–8 and PTAI-10–14 in mixtures with helper lipids in the in vitro model of XC rat Rous sarcoma cell line before using these formulations in vivo in rats. Plasmid DNA pEGFP‐C1 encoding reporter eGFP as well as plasmid DNA encoding mCherry reporter and VEGF-A protein were used to test in vitro efficiency of PTAI-based lipofecting mixtures. In our first experiments, we aimed at testing if efficiency of PTAI lipofecting mixtures is the same after preparation of lipoplexes in culture medium and 5% glucose as planned for in vivo experiments. It was an inevitable step as the composition of solution used for lipoplexes preparation can influence its formation, stability ,and effectiveness. The results showed no statistically significant differences between lipoplexes prepared in these two different solutions (Fig. 2a and b). Both compositions also showed the same pattern of slightly lower lipofection efficiency in the presence of serum compared to serum-free conditions (not statistically significant). Moreover, flow cytometry analysis was used to evaluate possible apoptosis signs after lipofection. Process of cell death causes changes in cellular morphology, resulting in cell shrinkage and fragmentation, which can affect scattering properties of cells analyzed by flow cytometry. In particular, due to the changes in cellular size and internal complexity, apoptotic cells typically possess decreased FSC signal, whereas their SSC signal increases. Thus, based on performed flow cytometry analysis of FSC and SSC parameters, we did not observe any significant changes in morphological characteristics of transfected cells that would indicate cell apoptosis (Supplementary Fig. S1).

In the second set of in vitro trials, we have tested the stability of lipoplexes at 37 °C in the period of 7 days as planned for intrarenal infusion with osmotic pumps. Lipoplexes were prepared in 5% glucose solution. The results showed higher efficiency of transfection for PTAI-10–14 composition (Fig. 2c, Supplemental Fig. S2) than PTAI-6–8. Both PTAI-6–8 and PTAI-10–14 compositions resulted in formulation of lipoplexes that were stable at 37 °C for 7 days (Fig. 2c, Supplemental Fig. S2). PTAI-10–14-based composition showed high transfection efficiency both—in the absence and presence of serum, while PTAI-6–8-based formulation was more efficient in serum-free conditions than in the presence of serum. In this set of experiments, manual counting of cells with fluorescent microscope was used. Additionally, the efficiency of PTAI-10–14-based composition stored at 37 °C was tested on the first day (24 h after preparation of lipoplexes) and on the 7th day by ImageStream® technology. The stability of lipoplexes (Fig. 2d, e) was confirmed, hence on the basis of the obtained results and our previous findings, which proved the activity of PTAI-6–8 and PTAI-10–14-based compositions in vivo (Stachyra et al. 2017), both formulations were chosen for further experiments.

### In Vivo

#### Intravenous Injection

**Fig. 3:**
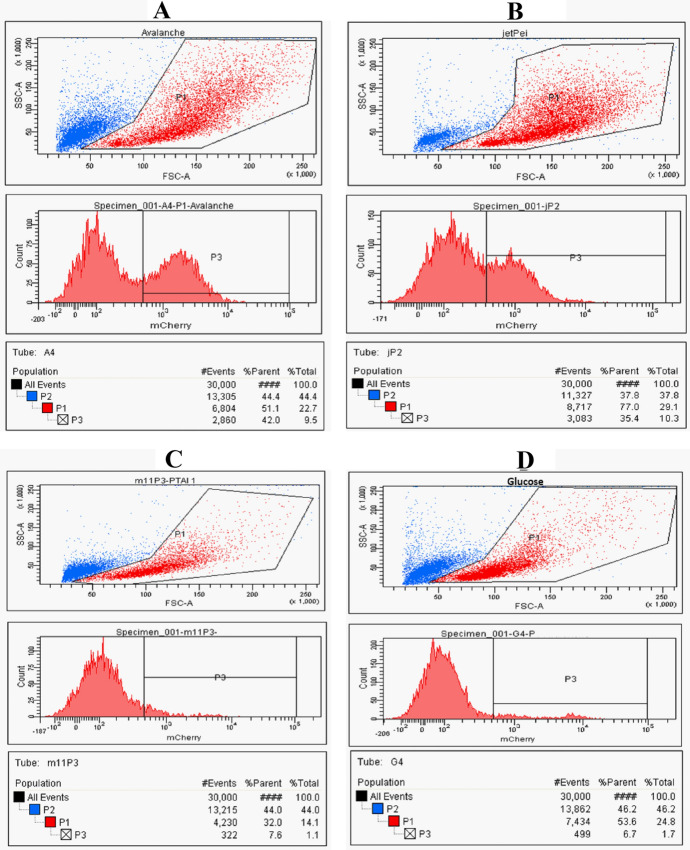
Representative flow cytometry analysis of fluorescence of mCherry in liver tissue homogenized to single cell suspension after intravenous injection of lipofection mixtures containing plasmid DNA mixed with different reagents for transfection **a** Avalanche®; **b** in vivo-jetPEI®; **c** PTAI-10–14; **d** 5% glucose (control group)

In the preliminary study, we wanted to compare selected reagents based on PTAIs to commercial carriers for transfection and to choose the optimal conditions for further experiments. Unfortunately, we were not able to obtain satisfactory results with any tested reagents. The fluorescence resulting from the expression of the reporter gene (eGFP or mCherry) was undetectable in almost all tissues (regardless of the different conditions and reagents tested; data not shown). Only in the liver, there were traces of reporter protein fluorescence (Table 1) with commercially available reagents (Avalanche and jetPei®). The representative flow cytometry analysis for each group is depicted in Fig. 3a–d. Interestingly, there was no correlation between the amount of applied DNA with the efficiency of transfection. Similar level of reporter protein fluorescence in the liver was observed after one and two injections. However, the administration of 180 µg of DNA at once (during 10 min infusion) seemed to cause slight toxic effect on the rat. The animal was lethargic for few hours and porphyrin staining was observed around the nose and eyes of the rat, which might indicate stress or sickness. The toxic effect was transient and rats’ behavior and appearance returned to normal on the next day.

**Table 1:** Summary of the preliminary in vivo experiments in which various conditions were tested for transfection after intravenous administration (individual subjects)

Reagent	Reporter gene	µg DNA per injection	µl of reagent	DNA: reagent ratio	No of inject	Total DNA, µg	Duration, h	Fluorescence in the liver, % parent
Avalanche®	eGFP	150	27	0.18	1	150	48	29.8
		150	30	0.20	2	300	48	31.3
		150	18	0.12	2	300	48	34.3
	mCherry	150	30	0.20	1	150	48	**42.0 (A)**
		150	30	0.20	1	150	24	51.0
		150	30	0.20	2	300	48	39.8
		180	30	0.17	1	180	72	32.5
jetPei®	mCherry	150	21	0.14	1	150	48	33.4
		150	27	0.18	1	150	48	**35.4 (B)**
	–	–	27	–	1	–	48	13.6
PTAI -10–14	eGFP	150	90	0.60	1	150	24	8.2
	mCherry	150	90	0.60	2	300	48	**7.6 (C)**
		150	90	0.60	1	150	24	7.6
5% glucose	–	–			1	–	48	11.1
					2		48	17.7
					1		24	**6.7 (D)**
					1		24	15.4
					2		48	13.9
					1		72	14.5
					1		48	23.0
					1		48	16.4
PBS	–	–			1	–	24	12.2

Flow cytometry analysis of selected subjects (bolded) from each group is illustrated on Fig. 3a–d

#### Intrarenal Infusion with ALZET® Osmotic Pumps (2 Week Observation)

**Fig. 4:**
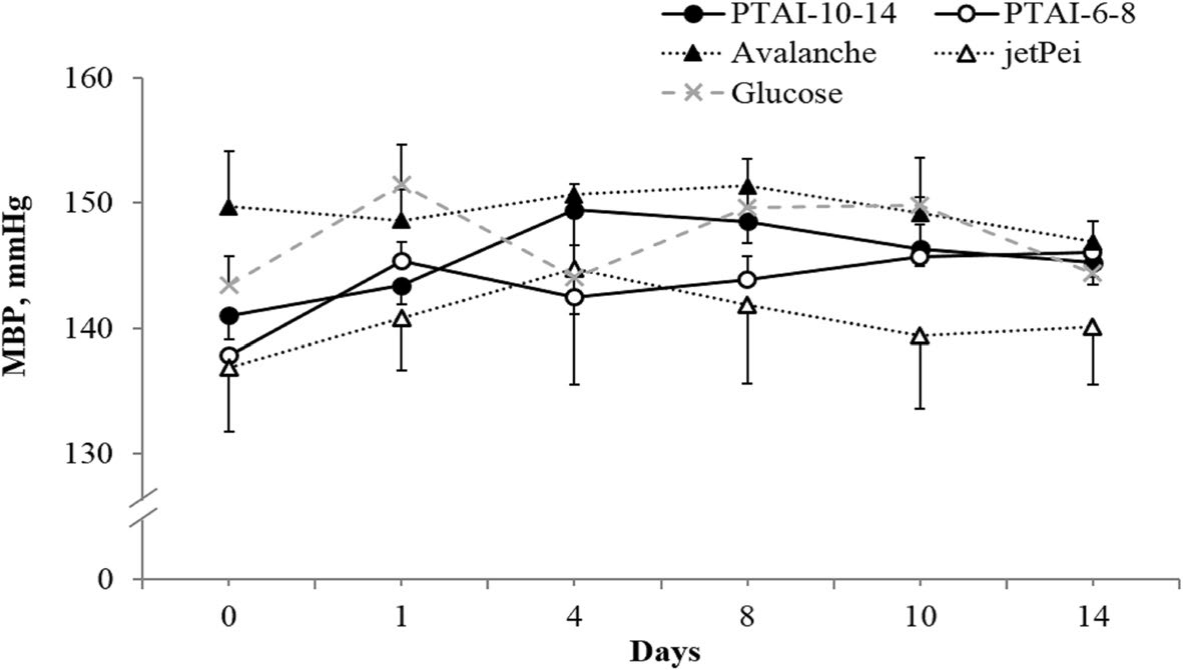
Mean blood pressure (MBP) of adult SHR receiving plasmid DNA encoding VEGF-A gene in osmotic pumps (model 2001) directly into the renal medulla mixed with different reagents for transfection: PTAI-10–14 (*n* = 4); PTAI-6–8 (*n* = 4); Avalanche® (*n* = 5); in vivo-jetPEI® (*n* = 5); 5% glucose (control group, *n* = 5); NS

Since intravenous delivery of DNA complexes was unsuccessful, we changed the route of administration to intrarenal. All rats were in a good health and no signs of toxicity were observed. All animals demonstrated normal weight gain which was not influenced by administered substances. Also diuresis, water, and food intake were similar in all groups and the values were within the normal range for adult SHR (data not shown). Mean blood pressure (MBP) was not affected during the 2 week observation (Fig. 4).

**Fig. 5:**
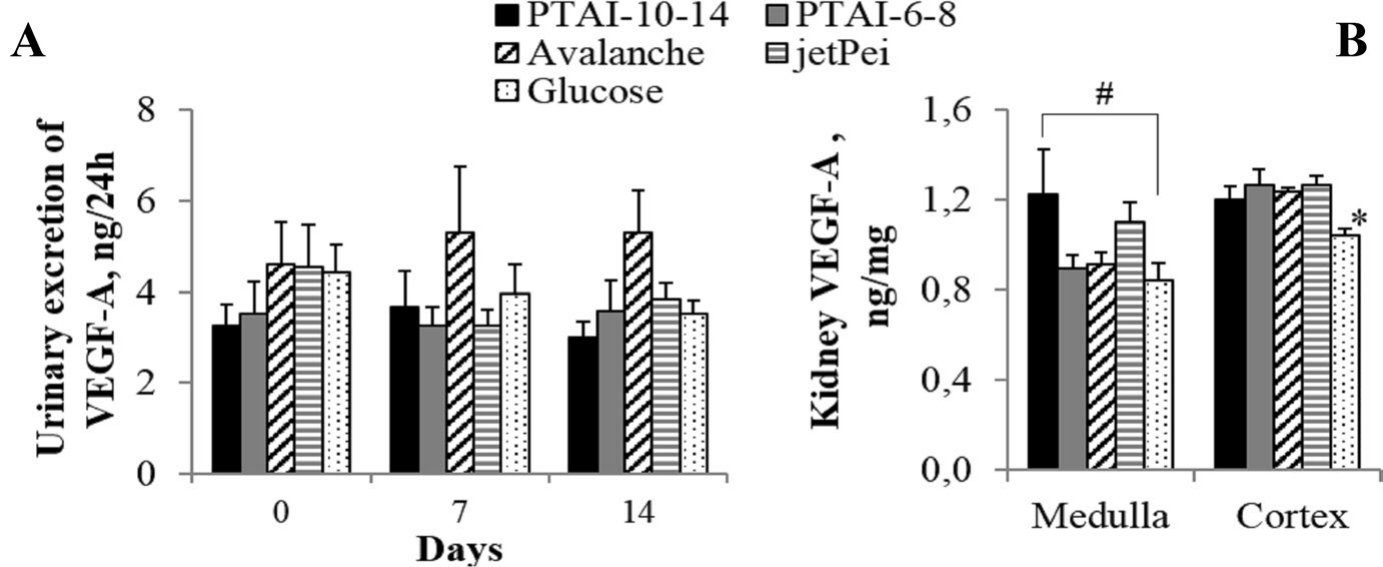
**a** Urinary excretion of VEGF-A and **b** VEGF-A level in the renal medulla and cortex of adult SHR receiving plasmid DNA encoding VEGF-A gene in osmotic pumps (model 2001) directly into the renal medulla mixed with different reagents for transfection: PTAI-10–14 (*n* = 4); PTAI-6–8 (*n* = 4); Avalanche® (*n* = 5); in vivo-jetPEI® (*n* = 5); 5% glucose (control group, *n* = 5); *p < 0.05 values in all groups *versus* control group (glucose); ^#^*p* < 0.05 PTAI-10–14 versus control group receiving glucose; one-way ANOVA

The level of VEGF-A was measured in urine and in renal homogenates (Fig. 5a and b). Only slightly higher urinary excretion of VEGF-A protein was observed after plasmid administration with Avalanche® reagent compared to other treatments; however, the difference was not statistically significant. The level of VEGF-A in the renal cortex was statistically higher in all groups receiving plasmid DNA with VEGF-A gene in comparison to control group, which received only solvent solution (5% glucose). In renal medulla, only after PTAI-10–14-DNA delivery, statistically higher level of VEGF-A protein was observed in comparison to control group which received glucose (Fig. 5b).

Flow cytometry analysis revealed no traces of mCherry fluorescence measured in single cell suspensions of homogenized kidneys. Results showed no differences between groups receiving plasmid DNA with different lipofectants (commercial and PTAI-based) and in comparison to control group which received only 5% glucose (data not shown).

## Discussion

Advances in gene delivery technology and progresses in understanding pathogenesis of various diseases resulted in implementation of the first clinical trials with therapeutic nucleic acids (Sridharan and Gogtay 2016). The major obstacle is the lack of efficient and safe DNA carriers (Ramamoorth and Narvekar 2015). Among the non-viral methods, lipofection is considered as one of the most promising option. Previous studies showed that new type of cationic lipids, called PTAI constitute a promising alternative in formulating new lipofecting agents. Lipofecting activity of PTAI allows to effectively transfect plasmid DNA into cells without significant side effects on cell physiology (Grecka et al. 2016; Rak et al. 2016; Stachyra et al. 2017). Moreover, compositions of PTAI and different co-lipids enable DNA and RNA transfer into a wide range of cell types both in the presence and absence of serum. Developed lipoplexes are non-toxic for rats (Gawrys et al. 2014a, b), are safe towards human RBSs and are effective DNA vaccines and drug carriers in vivo (Grecka et al. 2016; Rak et al. 2016; Stachyra et al. 2017; Gawrys et al. 2018). All these finding resulted in 3 received polish patents (PL 231158, 2019; PL 230096, 2018; PL 211824, 2012) and 1 international patent application (WO/2016/03234 8, PCT/PL2015/00 0093, pending) making PTAI an interesting choice in lipofection.

### In Vitro Efficiency and Stability Tests

The in vitro tests of PTAI-based lipofecting reagents were designed to evaluate if PTAI lipoplexes could be adjusted to in vivo administration in 5% glucose solution for single injections and for prolonged administration in osmotic pumps. Obtained results showing that 5% glucose solution is a suitable medium for PTAI-based lipoplexes preparation are in accordance with data previously shown in the literature that this solution is favorable for in vivo and in vitro nucleic acids delivery systems (Phua et al. 2013; Tao et al. 2016). Moreover, some data were also showing that glucose solution causes formation of smaller and more effective (especially in the presence of serum) lipoplexes (Sakaguchi et al. 2008). Additionally, glucose solutions are also recommended for in vivo transfections by producers of commercially available transfecting agents. In conclusion PTAI-based formulations can be prepared in 5% glucose exhibiting high efficiency of lipofection.

Stability of the formulation is a crucial issue, as lipoplexes structure protects DNA from degradation and determines the interactions with cell membrane. Lipoplexes stability in aqueous solutions is usually tested during hours (usually 4–5 h) after preparation—to ensure that they are stable within the time of incubation with cells. Commercially available lipofecting agents are stored in a form not mixed with nucleic acids and at lower temperature (4 °C). There are some trials to implement dehydration method for the storage of transfecting agents already complexed with nucleic acids (Kasper et al. 2011; Perche et al. 2012), which could be beneficial for many applications, such as storage of DNA vaccines. Nevertheless, high sensitivity of lipids and DNA to hydrolysis and oxidative degradation is underlined as the main obstacle in achieving stable and effective lipoplexes for prolonged storage (Varshney and Singh 2015). Thus, lipoplexes are usually only expected to be stable in cell culture medium for a few hours to interact with cell membrane in vitro or in the blood stream after in vivo injections. Testing stability of lipoplexes suspended in aqueous solutions and kept at 37 °C is unusual in literature. Planned delivery in osmotic pumps constituted a new challenge in the process of development of PTAI-based lipofectants. PTAI-based lipoplexes were proved to be stable not only in an aqueous solutions, but also at 37 °C for the period of 7 days, which constitutes a very promising option for prolonged drug delivery methods.

Usage of osmotic pumps is not common for DNA delivery. Some studies with osmotic mini-pumps showed that continuous intracerebral delivery of lipoplexes caused tumor regression (Samal and Dubruel 2015), which confirms the potential of osmotic pumps for targeted gene delivery. Demonstrated stability of PTAI-based lipoplexes makes them perfect candidates for future application with osmotic pumps without necessity of using additional, complicated steps, such as dehydration method. Results obtained in vitro and our previous findings (Grecka et al. 2016; Rak et al. 2016; Stachyra et al. 2017) gave us a good basis for in vivo experiments, ensuring administration of effective and stable PTAI-based lipoplexes.

### In Vivo

The goal of the first part of the in vivo study was to evaluate the effectiveness of PTAIs as DNA carriers, therefore we wanted to compare their efficiency to commercially available reagents for transfection. This part of the project proved to be more difficult than expected, because none of the applied reagents produced any satisfactory results. Out of two selected commercial reagents none provided stable and high expression of the reporter gene after intravenous administration. Both Avalanche®-in vivo Transfection Reagent and in vivo-jetPEI® are advertised as an extremely powerful reagents for in vivo delivery of any nucleic acid and to any animal model. Both products are based on cationic lipids or polymers, which according to the manufacturers assurances, should provide formation of the stable complexes with nucleic acids, protecting it from degradation and facilitating its delivery into the cells. For Avalanche® only a single successful report has been published so far describing the effect of *MYO3A* gene knockdown on breast cancer metastasis (Baghel et al. 2016). The authors injected female BALB/c mice intratumorally with *MYO3A-*directed shRNA. The injections were performed three times a week at a dose of 10 µg of shRNA mixed with 2 µL of the reagent in 50 µL of 5% glucose. In case of in vivo-jetPEI® the manufacturer assures on the product website that its proven track record is supposedly over 700 publications. In fact there is a “Polyplus-transfection Database”, which is quite accessible and easy to use. After selection of the in vivo-jetPEI® reagent and DNA as a type of nucleic acid there is 23 publications, which relate to intravenous infusion, however none of them in the rat. The majority of records describe studies in mice. Both manufacturers stipulate that the amount of nucleic acid must be determined according the administration route and the animal model and furthermore that the user need to optimize the amount of nucleic acids and its ratio to the reagent. Both product manuals include similar guidelines and starting conditions, which comprise of around 150 μg nucleic acid and 24–30 μl of the reagent in 1 ml of 5% glucose for intravenous infusion in the rat. In the present study we tried various conditions based on the given instructions (summarized in Table 1), unfortunately none produced any satisfactory results. Only in the homogenized liver there were some traces of the reporter protein fluorescence, achieved only with commercial reagents; however there was no correlation between the amount of applied nucleic acid, amount of injections or duration of the observation with the efficiency of transfection.

The observed results are somehow surprising, not only due to very low efficiency of all reagents, but also because there were no traces of fluorescence of the reporter gene in the lungs. All used reagents, commercial Avalanche® and in vivo-jetPEI®, as well as polyisoprenoid PTAI are cationic compounds, which should form complexes with nucleic acids, called lipoplexes (i.e., Avalanche® and PTAI) or polyplexes (in vivo-jetPEI®, which is built of linear polyethylenimine). It has been reported that lipoplexes are accumulated in the lungs after intravenous administration, due to electrostatic interactions between positive charges of complexes and negatively charged erythrocytes. These interactions may cause agglutination (particles clumping), which in turn might cause the entrapment of complexes in the lungs (Eliyahu et al. 2002; Simberg et al. 2003; Hattori et al. 2019). Due to this phenomena gene therapy for lung diseases lately gain significant importance (Kuruba et al. 2009; Merkel and Kissel 2012; McCaskill et al. 2013; Merkel et al. 2014). However it also has been reported that the lung uptake and the bio-distribution of the complexes after systemic delivery depend on numerous factors, especially the helper lipids used in the formulations (Liu et al. 1997; Sakurai et al. 2001).

Due to lack of satisfactory results after systemic administration we decided to change the route of administration from intravenous to intrarenal. We expected that such targeted therapy with much higher concentration of DNA administered chronically over prolonged period will turn out to be more fruitful for hypertension treatment. PTAI complexes were tested in the temperature of 37 °C and were proved to be stable and efficient after 7 days of incubation. Also, according to the manufacturers assurances, in vivo-jetPEI® can be used in osmotic pumps for chronic administration. This reagent was also used for intra-medullar administration in rats. Liu et al. (2008) used it for plasmid shRNA (100 μg) injection through catheter implanted into the renal medulla of male Sprague–Dawley rats. Moreover transfection with in vivo-jetPEI® in Dahl S rats was described too. Left kidney was transfected with plasmid DNA (50 μg) in combination with ultrasound treatment (Wang et al. 2010; Zhu et al. 2012). The authors claim that ultrasounds significantly enhance the efficiency of transfection with various reagents, including in vivo-jetPEI® (Hosseinkhani et al. 2005; Newman and Bettinger 2007; Li et al. 2008), while Polyplus-transfection support team assures that their reagent should work without ultrasounds. Unfortunately this also proved to be untrue at least in our hands. Flow cytometry analysis did not show any signs of reporter protein fluorescence in the kidneys.

Only application of PTAI-10–14 to deliver plasmid DNA resulted in higher medullary content of VEGF-A protein, which suggest its potential for in vivo gene delivery. However, in this group of rats (or any other) we did not observe any decrease in blood pressure. The level of expressed VEGF-A might be still too low to provoke the blood pressure change or its role in the control of blood pressure is not as crucial as initially suggested in this model of hypertension; however this question still needs to be answered in further studies.

## Conclusions

Conducted in vitro experiments showed that PTAI-based lipoplexes efficiently transfect XC rat sarcoma cells. Moreover, preparation of lipoplexes in 5% glucose solution for in vivo application (instead of cell culture medium for in vitro use) did not change the efficiency of DNA transfer. PTAI-based lipoplexes were also stable when stored at 37 °C for 7 days, which makes them suitable for prolonged DNA delivery in osmotic pumps.

After in vivo intravenous injection to SHR, only weak fluorescent signal of reporter protein mCherry for Avalanche® and jetPei® in the liver was observed. Prolonged 7-day intrarenal infusion with osmotic pumps resulted in slightly higher urinary excretion of VEGF-A for Avalanche® reagent (NS). Interestingly, VEGF-A level in renal medulla was significantly higher only for PTAI-10–14-based formulation.

In conclusion, despite the promising in vitro results, we were not able to achieve VEGF-A expression level high enough to verify VEGF-A gene therapy usefulness in SHR model. However, results of our study give important indications for the future development of PTAI-based DNA carriers and kidney-targeted gene delivery. Based on our research on three lipid-based and one polymer-based transfection reagents, we conclude that other factors such as ultrasounds or suction-mediated transfection (Taniguchi et al. 2016; Oyama et al. 2017) may be necessary to achieve efficient transfection in SHR model of hypertension.

## Electronic supplementary material

Below is the link to the electronic supplementary material.

Supplementary file1 (DOCX 2308 kb)
