# Supplementary material for: Polyprenol-Based Lipofecting Agents for In Vivo Delivery of Therapeutic DNA to Treat Hypertensive Rats
Source: Biochem Genet. 2020 Aug 6;59(1):62–82. doi: 10.1007/s10528-020-09992-9 (PMC7846535; doi:10.1007/s10528-020-09992-9)
Supplement: Supplementary file 1 — Supplementary file1 (DOCX 2308 kb) [file 10528_2020_9992_MOESM1_ESM.docx]

**SUPPLEMENTARY MATERIAL**

**Title: “Polyprenol-based lipofecting agents for *in vivo* delivery of therapeutic DNA to treat hypertensive rats”**

Olga Gawrys and Monika Rak, Iwona Baranowska, Sylwia Bobis-Wozowicz, Karolina Szaro, Zbigniew Madeja, Ewa Swiezewska, Marek Masnyk, Marek Chmielewski, Elzbieta Karnas, Elzbieta Kompanowska-Jezierska

Corresponding author:

Olga Gawrys; Department of Renal and Body Fluid Physiology, Mossakowski Medical Research Centre

5 A. Pawinskiego Street, 02-106, Warsaw, Poland; [olga.gawrys@gmail.com](mailto:ogawrys@imdik.pan.pl)


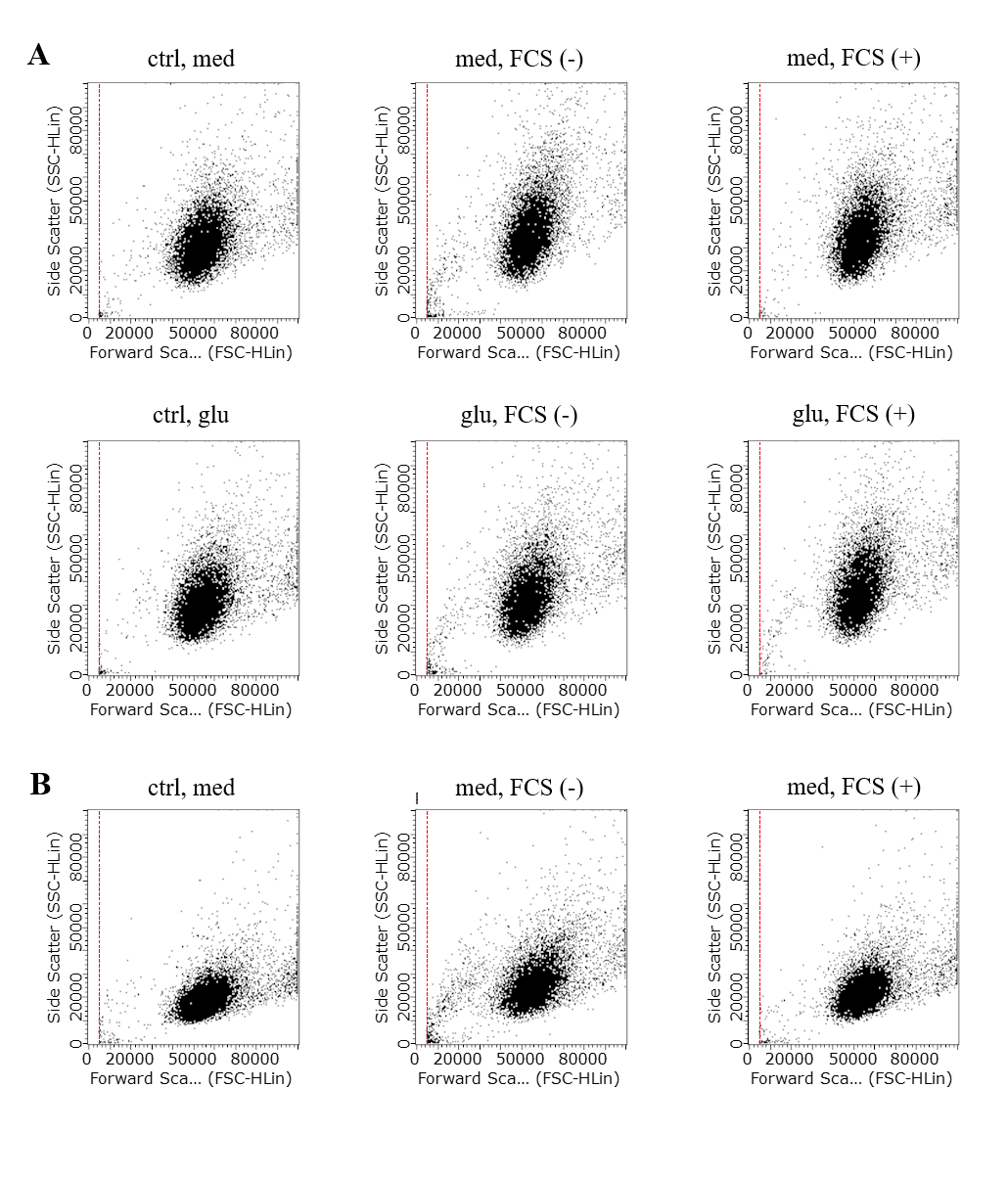


Supplementary Fig. S1

**Flow cytometry analysis of cell morphological features related to cell viability after DNA transfer into XC cells in the presence (FCS+) and absence (FCS-) of serum with PTAI-based lipoplexes.** XC cells (**A**) and DU145 cells (**B**) were transfected with PTAI-10-14 + DOPE using pEGFP-C1 plasmid. Lipofecting mixtures were prepared in cell culture medium (med) or 5 % glucose solution (glu) and used in the absence [FCS (-)] or presence [FCS (+)] of serum. Exemplary dot plots of forward scatter (FSC) vs. side scatter (SSC) parameters, corresponding to relative size vs. granularity of cells, respectively. Cells were acquired using Guava easyCyte flow cytometer and analyzed by Guava InCyte Software.


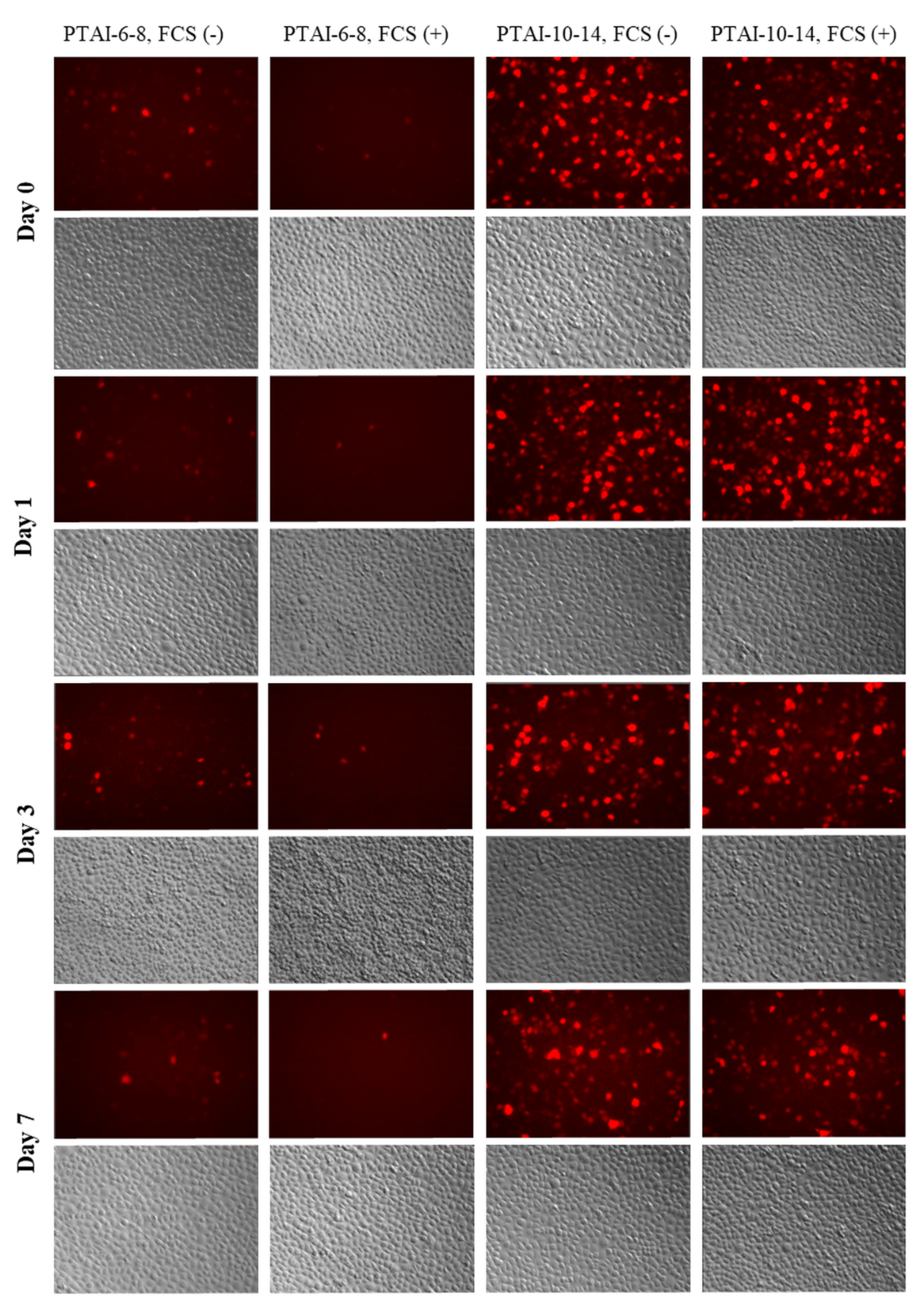


Supplementary Fig. S1

Efficiency of DNA transfer into XC cells in the presence (FCS+) and absence (FCS-) of serum with PTAI-based lipoplexes. Representative images of XC cells transfected with PTAI—6-8 + DOPE and PTAI-10-14 +DOPE+DC-cholesterol using mCherry-VEGF-A plasmid with lipoplexes prepared in 5% glucose and stored at 37°C for 1-7 days. Molar ratios: PTAI-6,7,8 + DOPE – 1.5:1 and PTAI-10-14 + DOPE + DC-cholesterol – 1:1:1, PTAI+DOPE concentration: PTAI—6-8-based lipoplexes - 4 µg/well of 24-well plate, PTAI-10-14-based lipoplexes - 4 µg/well of 24-well plate, DC-cholesterol added as additional lipid at indicated molar ratio
